# Determination of Strength Parameters of Composite Reinforcement Consisting of Steel Member, Adhesive, and Carbon Fiber Textile

**DOI:** 10.3390/ma17236022

**Published:** 2024-12-09

**Authors:** Maciej Adam Dybizbański, Katarzyna Rzeszut, Saydiolimkhon Abdusattarkhuja, Zheng Li

**Affiliations:** 1 Faculty of Civil and Transport Engineering, Poznań University of Technology, 5 Marii Skłodowskiej-Curie Str., 60-965 Poznań, Poland; katarzyna.rzeszut@put.poznan.pl (K.R.); saydiolimkhon.abdusattarkhuja@put.poznan.pl (S.A.); 2 Institute of Civil Engineering, Technische Universität Berlin, G.-Meyer-Alle 25, 13355 Berlin, Germany; dr.zheng.li@web.de

**Keywords:** adhesive joints, composite fabric, cold-formed steel elements, numerical simulation, cohesive zone model, laboratory tests

## Abstract

The main aim of the study was the determination of the strength parameters of composite bonded joints consisting of galvanised steel elements, an adhesive layer, and Carbon-Fiber-Reinforced Plastic (CFRP) fabric. For this purpose, shear laboratory tests were carried out on 60 lapped specimens composed of 2 mm thick hot-dip galvanised steel plates of S350 GD. The specimens were overlapped on one side with SikaWrap 230 C carbon fibre textile (CFT) using SikaDur 330 adhesive. The tests were carried out in three series that differed in overlap length (15 mm, 25 mm, and 35 mm). A discussion on the failure mechanism in the context of the bonding capacity of the composite joint was carried out. We observed three forms of joint damage, namely, at the steel-adhesive interface, fibre rupture, and mixed damage behaviour. Moreover, an advanced numerical model using the commercial finite element (FE) program ABAQUS/Standard and the coupled cohesive zone model was developed. The material behaviour of the textile was defined as elastic-lamina and the mixed-mode Hashin damage model was implemented with bi-linear behaviour. Special attention was focused on the formulation of reliable methodologies to determine the load-bearing capacity, failure mechanisms, stress distribution, and the strength characteristics of a composite adhesive joint. In order to develop a reliable model, validation and verification were carried out and self-correlation parameters, which brought the model closer to the laboratory test, were proposed by the authors. Based on the conducted analysis, the strength characteristics including the load-bearing capacity, failure mechanisms, and stress distribution were established. The three forms of joint damage were observed as steel-adhesive interface failure, fibre rupture, and mixed-damage behaviour. Complex interactions between the materials were observed. The most dangerous adhesive failure was detected at the steel and adhesive interface. It was also found that an increase in adhesive thickness caused a decrease in joint strength. In the numerical analysis, two mechanical models were employed, namely, a sophisticated model of adhesive and fabric components. It was found that the fabric model was very sensitive to the density of the finite element mesh. It was also noticed that the numerical model referring to the adhesive layer was nonsensitive to the mesh size; thus, it was regarded as appropriate. Nevertheless, in order to increase the reliability of the numerical model, the authors proposed their own correlation coefficients α and β, which allowed for the correct mapping of adhesive damage.

## 1. Introduction

The phenomenon of adhesion is fundamental to many applications across scientific disciplines, from the development of advanced adhesives in engineering to the evolutionary adaptations seen in nature. Understanding the intricate interplay in adhesion forces is crucial for optimising these applications and enhancing material performance [1]. The complexity in adhesion mechanisms not only highlights the importance of physicochemical interactions but also underscores the diversity that strategies organisms employ to navigate their environments [2].

**Figure 1:**
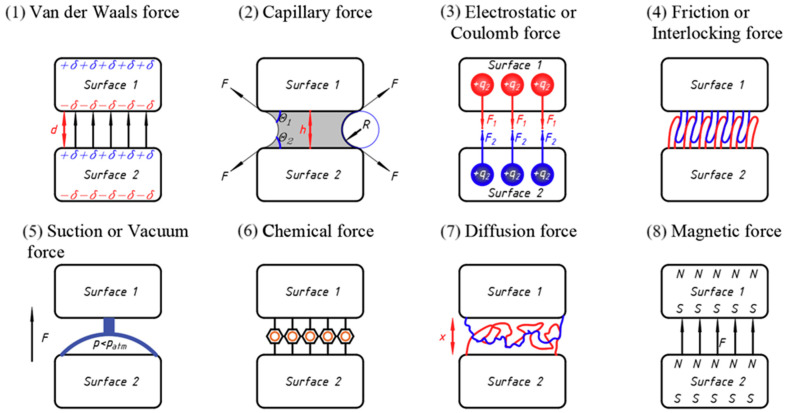
Schematic illustration of the mechanisms of adhesion and the various forces that exist between two surfaces in nature. (1) Van der Waals force; (2) capillary force; (3) electrostatic or coulomb force; (4) friction or interlocking force; (5) suction or vacuum force; (6) chemical force; (7) diffusion force; (8) magnetic force [3].

A profound comprehension of adhesion forces and their underlying physical properties might provide a suitable framework for the design of bonded joints [3]. Figure 1 represents the most notable and common physicochemical forces found in nature and illustrates their interactions between two surfaces, which used to be called adhesion. The interpretation of the term “adhesion” differs across various domains of research, including chemistry, biology, physics, and engineering. Surface forces are the fundamental interactions that facilitate the convergence of two bodies, while adhesion refers to the energy expended to maintain their connection.

Figure 1 delivers a thorough and intricately detailed picture that captures a variety of adhesion mechanisms and forces, all of which are notably derived from the complexities of the natural ecosystem, with particular attention to the surprising adaptations and capabilities exhibited by different materials on slippery surfaces. Herein lie the crucial and salient details concerning the diverse adhesion mechanisms and the corresponding forces that are vividly depicted in the aforementioned figure:

Van der Waals forces—These are weak attractive forces that occur between molecules. In the context of adhesion, van der Waals forces play a significant role in the adhesion of geckos [4] and other creatures to surfaces. Figure 1 illustrates how these forces contribute to the overall adhesion when the animal’s foot comes into contact with a surface [5].

In rigid materials, van der Waals forces contribute to adhesion by creating attractive interactions between surfaces, as demonstrated in the analysis of a rigid sphere and a half-elastic region [6]. In biological systems, van der Waals forces enhance cell adhesion, particularly in stem cells exposed to cold atmospheric plasma, which modifies the forces at play between cells and their extracellular matrix [7].

Capillary action, which can be defined as the ability of a liquid to flow in narrow spaces without the assistance of external forces, stands as a pivotal mechanism that significantly contributes to the phenomenon of adhesion, particularly under conditions characterised by the presence of moisture. The illustration provided serves to elucidate the manner in which the moisture present within the microstructures found in the feet of various animals, such as those exhibited by frogs, effectively enhances the process of adhesion through the engagement of capillary forces that operate at a microscopic level. This particular mechanism facilitates an improved ability to maintain grip on surfaces that are inherently slippery by drawing water into minuscule interstices, thereby substantially increasing the overall area of contact between the adhering surfaces [8].

Electrostatic forces also play an important role in adhesion. Figure 1 shows how positively charged amino acids in bristle surfaces promote adhesion through electrostatic interactions, enhancing their ability to adhere to different surfaces.

Friction or interlocking force refers to a type of adhesion where one surface adheres to another surface mechanically, increasing adhesion due to its physical structure. This structural adaptability allows animals living in appropriate natural conditions to move on slippery surfaces [9].

The suction force is generated when there is a pressure difference between the inside of a cavity (like a suction cup) and the external environment. This difference creates a vacuum effect that allows the suction cup to adhere to surfaces. The force can be mathematically expressed as follows:

*F* = *p ⋅ A,* (1)

where *p* is the pressure outside the suction cup (typically atmospheric pressure) and *A* is the area of the surface covered by the cup. This equation indicates that the suction force increases with the area of contact and the pressure differential.

The suction force works on the principle of creating a low-pressure zone inside a cavity. For instance, when an octopus uses its suckers, it contracts its muscles to expel liquid from the cavity, reducing the internal pressure and creating suction. This process allows the octopus to adhere to surfaces effectively [10].

Chemical forces arise from the interactions between molecules, primarily through bonds such as hydrogen bonds, covalent bonds, and ionic bonds. These forces are crucial for the adhesion between surfaces, influencing how materials stick together or resist separation.

Diffusion force refers to the movement of particles from an area of higher concentration to an area of lower concentration. This process is driven by the concentration gradient and is essential in various biological and physical systems, including adhesion mechanisms on slippery surfaces.

Magnetic force arises from the interaction between magnetic fields and charged particles. It can either attract or repel depending on the polarity of the magnetic charges involved. This force is crucial in various applications, including the design of materials that can enhance grip on slippery surfaces [11].

Speaking about the methods of investigating van der Waals forces, which are described above, we should note the measurement method shown in the article [12]. Advanced techniques such as interferometric force spectroscopy integrated with scanning electron microscopy allow for the measurement of van der Waals forces at sub-nanonewton resolutions, isolating these forces from other interactions [13].

In terms of the stress state of a steel plate, [14] outlines a significant advancement in assessing the mechanical stress state of steel plates using eddy currents and artificial intelligence (AI) techniques, aiming for high accuracy in stress mapping and evaluation.

It should be emphasised that the phenomenon of adhesion represents an exceedingly intricate, difficult, and complex problem. In engineering practices, it is postulated that the failure of a joint should not be of an adhesive nature, and in order to achieve that goal, it is needed to perform the appropriate preparation of the surfaces of the components. Reference [15] covers the developments in failure mechanics and the extended finite element method within the finite element system, supported by application examples. In addition, [15] presents classical methodologies of continuum mechanics and fracture mechanics and discusses boundary element and finite difference methods with an indication of their optimal applications. Also, [16] discusses the development of a stochastic model to analyse the behaviour of adhesive joints. This method aims to address the uncertainties in material properties and loading conditions that can affect the performance of these joints. By incorporating stochastic methods, the model can predict variability in the joint’s response, providing a more comprehensive understanding of its behaviour under different conditions. This approach helps in designing more reliable and efficient adhesive joints for various engineering applications. Unfortunately, currently, there is no numerical method that universally solves all questions. However, the cohesive zone model (CZM) is useful for analysing adhesive joints. The widely recognised cohesive zone model (CZM) is predicated on the simulation of failure characterised by cohesion, specifically in scenarios where a fracture propagates within the adhesive layer. CZM has become the predominant methodology for investigating the dynamic behaviour of adhesive joints such as fatigue, variable strain rate, and impact. Although, in general, the dynamic behaviour of adhesive joints is divided into three areas: fatigue, variable strain rate and impact, and modal analysis [17]. Therefore, this model is very useful in the case of engineering analysis. Grounded in the principles of the CZM, the authors attempted to establish a model that accurately reflects adhesive failure at the adhesive–steel interface, which was also empirically observed in laboratory conditions.

The research presented in this paper concerns the formulation of reliable methodologies to determine the strength characteristics of a composite reinforcement consisting of the steel element, adhesive, and CFRP fabric. This issue of the investigation covers the characterisation and evaluation of an adhesive interface between galvanised steel and CFRP fabric with the aim of assessing its mechanical attributes, which include the load-bearing capacity of the bond, failure mechanisms, and stress distribution. Strength characteristics will be established through a synthesis of existing literature, numerical modelling, and empirical laboratory experimentation.

## 2. Experimental Tests

### 2.1. Problem Formulation

**Figure 2:**
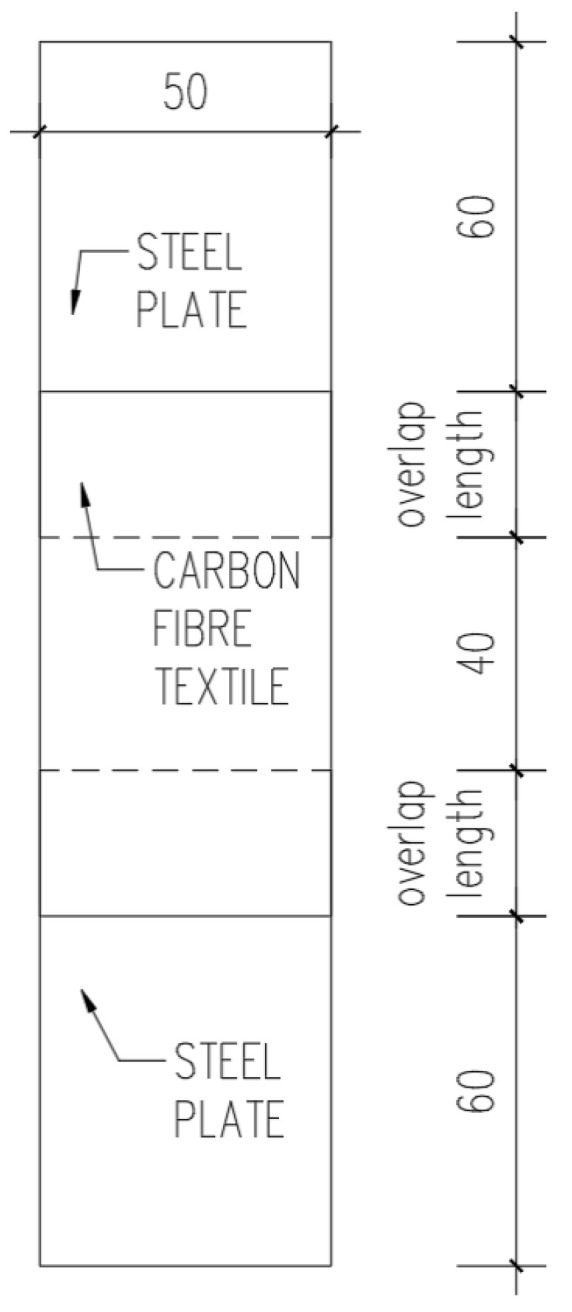
Schematic of the sample.

In the first part of the research, a lapped shear laboratory test was performed. The specimens were made from steel plates (2 mm thick) overlapped on one side with SikaWrap 230 C [18] carbon fibre textile (CFT) using SikaDur 330 [19] adhesive. Both SikaWrap 230 C and SikaDur 330 were provided by Sika Poland sp. z o.o. (Warsaw, Poland). The tests were carried out on 60 specimens, 3 series (A, B, and C) with 20 specimens each. For each series, a different overlap length was considered, namely 15 mm, 25 mm, and 35 mm (Table 1). The samples were produced as shown in Figure 2.

Before creating a bonding connection, the steel plates’ thicknesses were measured. The steel plate surface was cleaned with sandpaper and degreased with acetone. After surface preparation, the adhesive layer was applied uniformly on the steel plate surface, followed by one layup of CFT. The CFT was pressed to the steel surface, creating a bonded connection and embedding the carbon fibres in the epoxy matrix. Moreover, the excess of adhesive was removed from the surface of the textile. After the hardening process of the adhesive was finished, which took 7 days, the measurement of each single lap joint (SLJ) geometry was performed. The difference between the second and first measurements, reduced to a CFT thickness of 0.129 mm, was the adhesive thickness.

**Figure 3:**
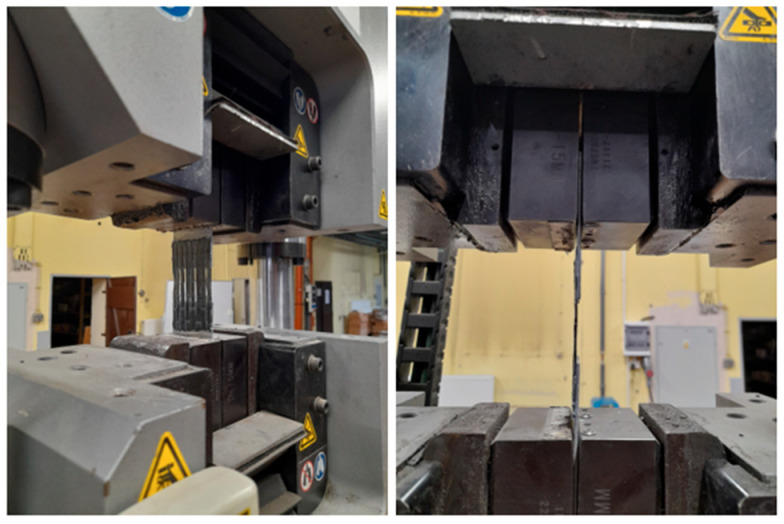
Specimen in the grips of a testing machine.

For experimental testing, an INSTRON SATEC 300 DX (Instron Polska, Opole, Poland) testing machine was used. The load accuracy measurement was 0.6 kN and the displacement accuracy was 0.13 mm. The testing machine was set to zero after the test loading system was assembled, but before the specimen was actually gripped at both ends. Once the force zero point was set, the force measurement system was not altered in any way during the test. The tensile tests were carried out under displacement control conditions. The samples were stretched at a speed of 0.01 mm/s until the specimens failed. An example of the prepared specimen in the grips of the testing machine is shown in Figure 3. After the completion of the laboratory tests, an observation of the nature of the fracture for all adhesive SLJs was conducted.

### 2.2. Experimental Test Results

The test results for series A, B and C are summarised in Table 2, Table 3 and Table 4. The mean value of the maximum load and the corresponding imposed displacement were reported for the respective series of the specimens. The standard deviation was determined for each parameter.

**Figure 4:**
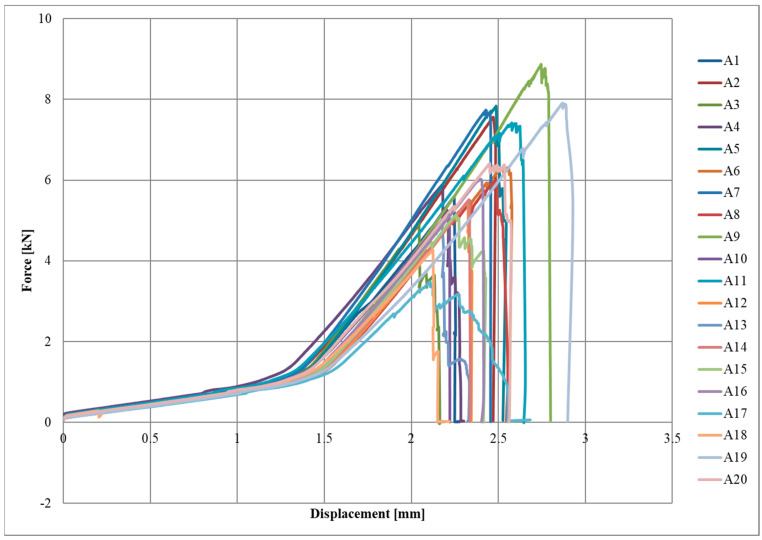
Force-displacement diagram for A series.

**Figure 5:**
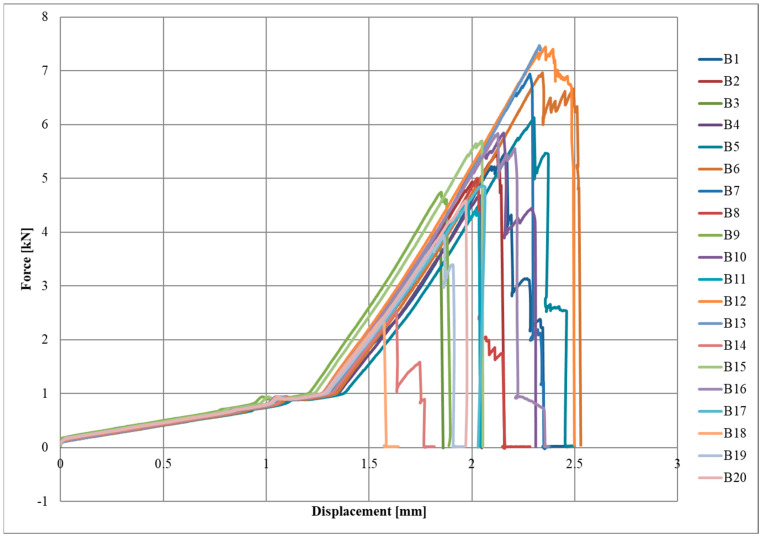
Force-displacement diagram for B series.

**Figure 6:**
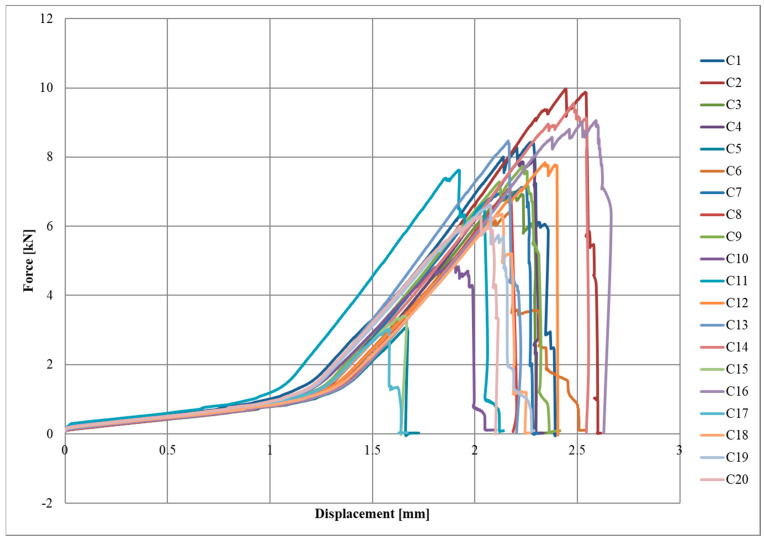
Force-displacement diagram for C series.

Diagrams of the force–displacement relationships for each series, A, B, and C, are shown in Figure 4, Figure 5 and Figure 6, respectively.

The initial slow increase in force due to the lower initial elastic modulus of the textile can be observed in the graphs. Once a displacement of approximately 1.1–1.5 mm was reached, there was a sudden increase in force, which was a straight line until the specimen broke. The destruction of the joint occurred rapidly or gradually, with successive fragments of CFRP textile breaking off of the steel surface.

**Figure 7:**
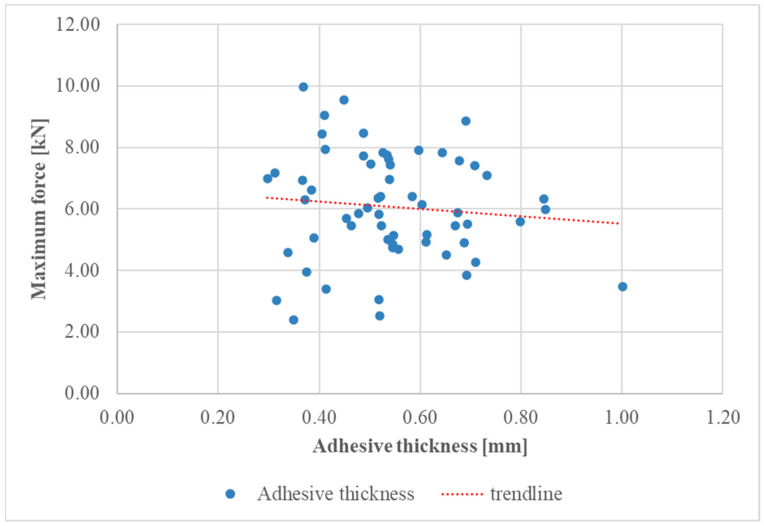
Maximum force-adhesive thickness diagram for all series.

**Figure 8:**
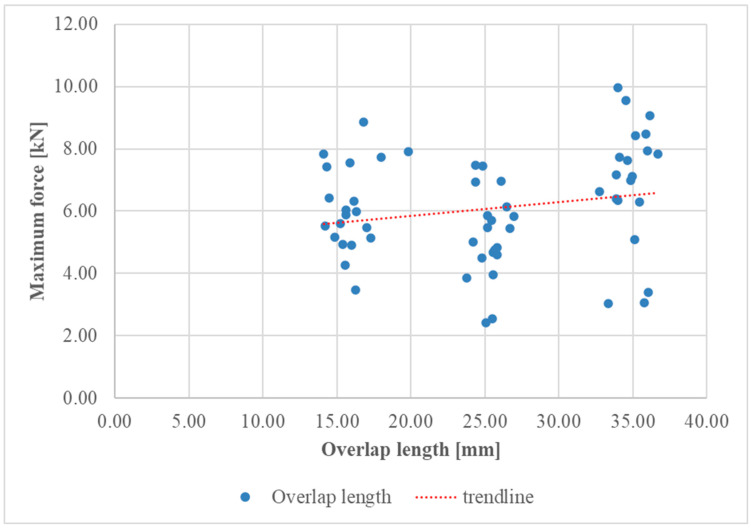
Maximum force-overlap length diagram for all series.

Diagrams of the maximum force–overlap length and maximum force–adhesive thickness relationships for the test specimens are shown in Figure 7 and Figure 8, respectively.

The data points in Figure 7 represent various specimens with differing adhesive thicknesses. The observed trend indicated that greater adhesive thickness resulted in a decreased maximum force capacity. This implies that thicker adhesive layers may compromise joint strength, potentially due to defects within the adhesive.

Meanwhile, Figure 8 shows the relationship between the maximum force applied to the specimens and the overlap length of the bonded joints, and the data points represent different series of specimens with varying overlap lengths. The diagram reveals that as the overlap length increased, the maximum force that the joint could withstand also increased. This indicates that a longer overlap length enhanced the strength of the bonded joint, likely due to the larger bonded area distributing the applied load more effectively and reducing stress concentrations.

### 2.3. Observation of Connection Structure Damage

**Figure 9:**
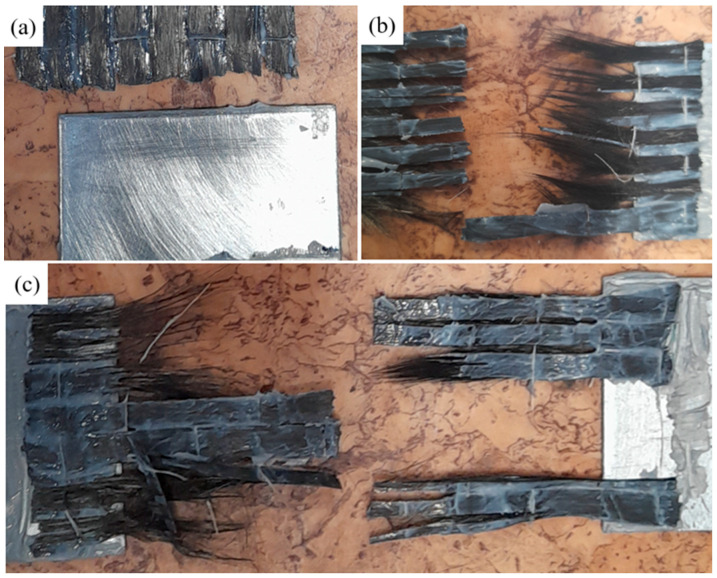
Observed forms of damage: (**a**) adhesive failure at steel/adhesive interface, (**b**) fabric rupture, (**c**) mixed failure.

Observations of the nature of fracture for CFRP-Steel adhesive connection were conducted. A visual inspection was carried out. Figure 9 shows examples of damaged samples.

Upon visual inspection of the damaged samples, different types of damage were observed. For some samples, damage was only observed at the steel/adhesive interface, and for others, fibre rupture. Others were distinguished by mixed damage behaviour. However, in none of the samples, cohesive failure in the adhesive layer or delamination was observed. A full description of the failure mechanisms for each sample is presented in Table 5, where A denotes adhesive failure at the steel/adhesive interface and F corresponds to fabric rupture.

Table 6 presents the average strength of the joint and its standard deviation for each type of failure in different test series.

**Figure 10:**
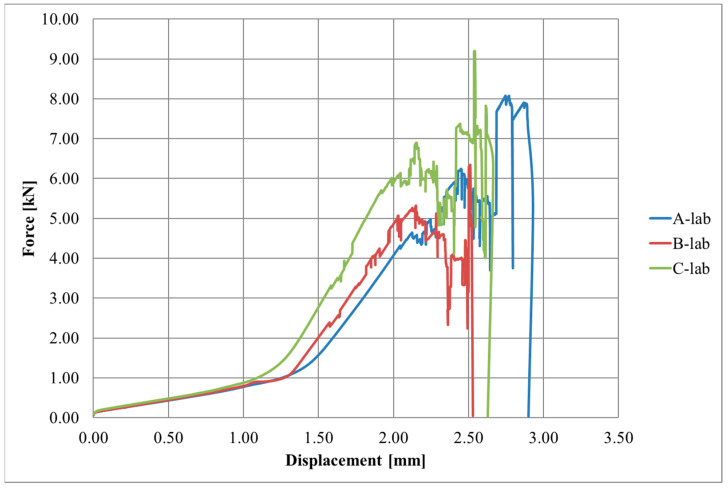
Average force-displacement diagrams for each series—lab.

Figure 10 presents the average force–displacement diagrams for each series.

It should be noted that the joint stiffness and joint strength increased proportionally to the overlap length. Moreover, an increase in the adhesive thickness caused a decreased joint strength. It was also observed that the adhesive failure occurred at the steel interface, indicating that the bond between the steel and adhesive was a critical point. Simultaneously, there was a combination of adhesive failure and fabric rupture, suggesting complex interactions between the materials.

## 3. Numerical Analysis

A numerical analysis was conducted in the Abaqus program and later verified and validated based on laboratory test results. Two models were developed, referring to damage mechanisms (Table 5). In the first stage, the goal was to define the material parameters of carbon fibre textile. Second, it was to define the cohesive zone model (CZM) for adhesive connection. The relevant computer hardware and the system type used are presented in Table 7.

**Figure 11:**
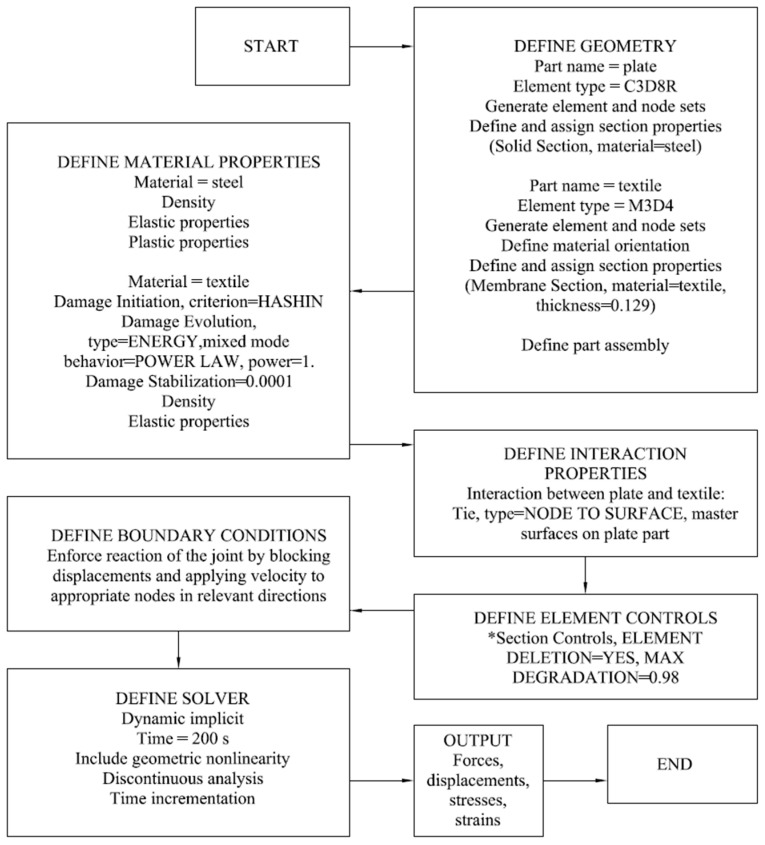
Block diagram for numerical model of textile.

**Figure 12:**
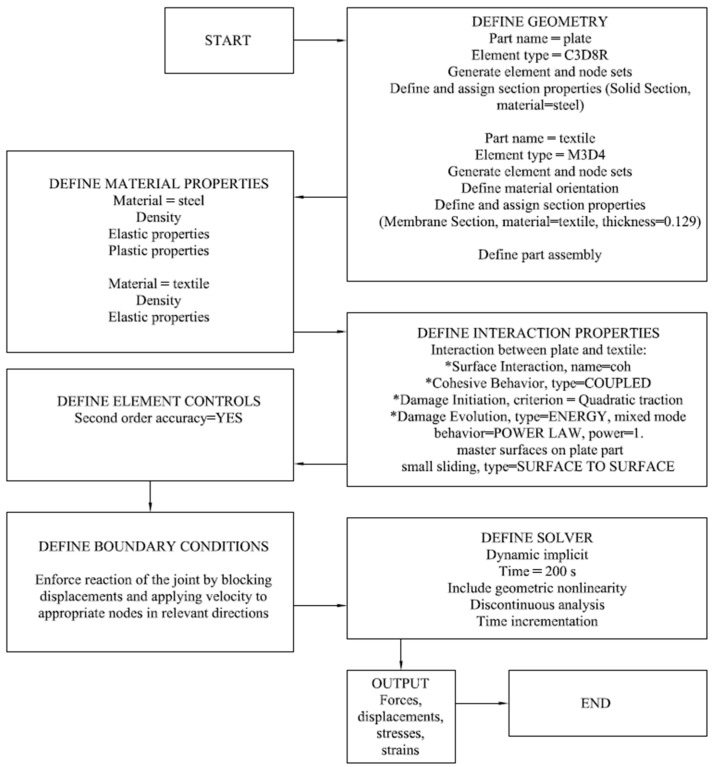
Block diagram for numerical model of adhesive.

Figure 11 and Figure 12 detail the steps for setting up a Finite Element Analysis (FEA) simulation for steel and textile adhesive connection. The diagrams outline the process from defining material properties to obtaining output results.

### 3.1. Numerical Model—Fabric Rupture

ABAQUS [20], a commercial finite element program, was used to simulate the fabric rupture mechanism. The elastic material properties of the carbon fibre textile were taken from [21] with elastic properties given in Table 8. The material behaviour of the textile was defined as elastic–lamina, and the mixed-mode Hashin damage model [22] was implemented with bi-linear behaviour. That model is based on criteria that consider four different damage initiation mechanisms in the form of fibre tension, fibre compression, matrix tension, and matrix compression. The general concept of damage in unidirectional lamina is characterised by the degradation of material stiffness. It holds significant importance in understanding fibre-reinforced composite mechanics. Numerous such materials demonstrate elastic–brittle characteristics; in particular, the initiation of damage in these materials occurs without considerable plastic deformation. Therefore, the influence of plasticity can be dismissed when assessing the behaviour of these materials. It is assumed that the fibres within the fibre-reinforced composite are arranged in a parallel orientation.

Based on the current research reports, four distinct failure mechanisms appear: tensile fibre rupture, compressive fibre buckling and kinking, transverse tensile and shear-induced matrix cracking, and transverse compressive and shear-induced matrix crushing. The analysis in ABAQUS utilised the failure initiation criteria proposed by Hashin and Rotem [23] as well as Hashin [24], which express the failure surface in the effective stress space. In this model, longitudinal strength was taken from manufacturers data [18], and the remaining parameters were identified during the verification and validation of the model based on the laboratory tests.

**Figure 13:**
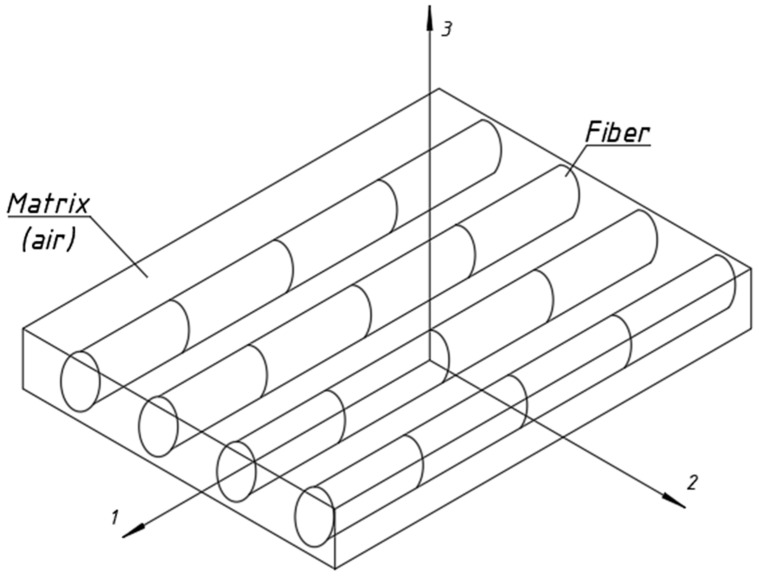
The ABAQUS anisotropic damage model, which is based on research conducted by Matzenmiller et al. [24], Hashin and Rotem [22], Hashin [23], and Camanho and Davila [25].

After the homogenisation of the fabric, it was assumed that the fibres were contained within the matrix as defined by the Hashin model, with the material properties given in Table 9. However, it should be pointed out that in this analysis, the matrix was modelled as air (Figure 13).

Given that the matrix was air, the transverse characteristics should be equal to “0”; nevertheless, this will result in dividing by “0”, and the load bearing capacity of the fabric would be equal to infinity. For this reason, these characteristics must be as small as possible; however, it is necessary to validate the model based on the laboratory results. The value of the parameters was defined as 5 MPa, which was equal to 0.16% of the longitudinal strength of 3200 MPa.

**Figure 14:**
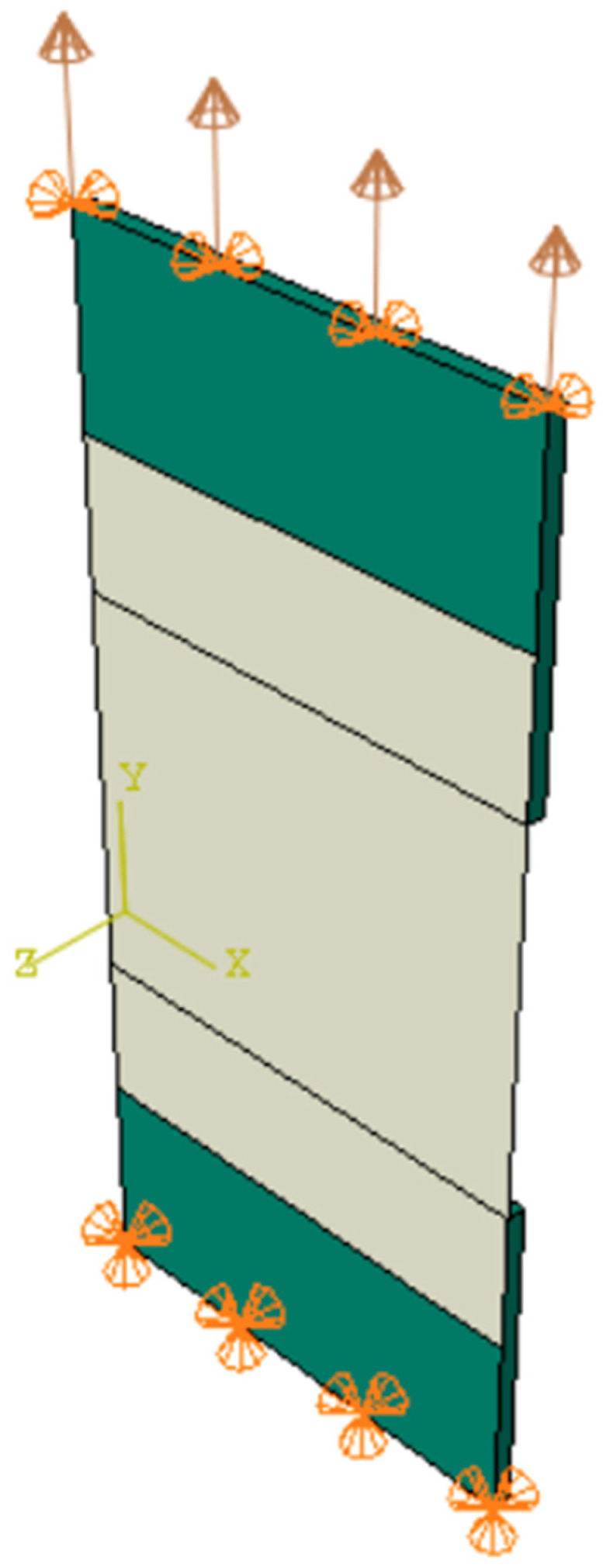
Applied boundary conditions.

The calculations were conducted using the dynamic implicit procedure, accounting for geometrical nonlinearity and a time period of 200 s. The boundary conditions applied are presented in Figure 14. On the bottom edge, the displacements were all blocked. Similarly, on the top edge, the displacements were blocked, except the vertical displacement. The displacement velocity was set to 0.01 mm/s in order to enforce tension. Tie connection was used between the plate and textile.

An extended mesh sensitivity analysis was performed. Moreover, the impact of the desired material properties (considering the Hashin damage definition) was included. The individual cases are summarised in Table 10.

### 3.2. Numerical Model—Adhesive Connection

In engineering practices, it is assumed that the destruction of the connection should not be of an adhesive nature. Therefore, in order to prevent adhesive destruction, it is necessary to properly prepare the surfaces of the joined elements. On the other hand, the commonly known cohesive zone model (CZM), as the name suggests, allows for the simulation of cohesive destruction, i.e., in which the crack develops in the adhesive layer. Based on the CZM, the authors of this paper attempted to determine a model that would correspond to adhesive destruction at the adhesive–steel interface, which was observed in the laboratory. The ABAQUS program was used to implement a coupled CZM. Given the extremely thin adhesive layer, local effects within the layer were disregarded. A surface-based cohesive behaviour was utilised, characterised by a mixed-mode cohesive law, using the power law criterion. By constructing traction–separation behaviour with coupled and specified stiffness coefficients, the cohesive behaviour was defined. The quadratic traction criterion was used to define the damage initiation criterion, and an energy type with linear softening behaviour was identified as the damage evolution behaviour. We can characterise the initial stiffness matrix as follows:

(2) $$K=\left[\begin{array}{ccc} K_{nn} & 0 & 0\\ 0 & K_{ss} & 0\\ 0 & 0 & K_{tt} \end{array}\right]=\left[\begin{array}{ccc} 90 & 0 & 0\\ 0 & 35 & 0\\ 0 & 0 & 35 \end{array}\right][MPa/mm]$$

where $K_{nn},\;K_{ss},\;and\;K_{tt}$ are the elastic stiffnesses in the normal and the two-shear directions, respectively. $K_{nn}$ is equal to the initial slope of the traction–separation model for mode I and can be described as follows:

(3) $$K_{nn}=\alpha \frac{E_{a}}{T_{a}}$$

where:

α—a parameter proposed by the authors to bring the destruction mechanism closer to that observed in the laboratory, α=0.01 [−];

$E_{a}$—tensile elastic modulus of an adhesive;

$T_{a}$—thickness of the adhesive.

Both $K_{ss}$ and $K_{tt}$ are the same and are equal to the initial slope of the traction–separation model for mode II loading and can be described as follows:

(4) $$K_{ss}=K_{tt}=\alpha \frac{G_{a}}{T_{a}}$$

where:

α—a parameter proposed by the authors to bring the destruction mechanism closer to that observed in the laboratory, $\alpha =0.01\;\left[-\right];\;$$G_{a}$—shear modulus of an adhesive;

$T_{a}$—thickness of the adhesive.

It was assumed that surface preparation, which affects adhesive failure, has the same impact on the elastic stiffnesses in all directions. For this reason, the correction factor in formulae 3 and 4 is the same. The stiffness reduction was intended to simulate the uniform distribution of stresses along the length of the adhesive and, consequently, the sudden development of joint failure. The damage properties of cohesive surface are presented in Table 11. As mentioned, the CZM refers to the failure inside the adhesive. To simulate the adhesive failure, a reduction parameter of β=0.2 [−] was proposed. The maximum nominal stress in all directions was defined as follows:

(5) $$t_{n}=t_{s}=t_{t}=\beta R$$

where:

$t_{n},t_{s},\;t_{t}$—normal and shear tractions in each direction;

R—the tensile strength of the adhesive given by the manufacturer, *R* = 30 MPa.

The symbol in square brackets [−] indicates that the parameters α and β are dimensionless.

**Table 11:** Cohesive surface properties implemented in FEM model.

Parameter	Quantity	Unit
Maximum nominal stress in all directions	6	MPa
Normal fracture energy	0.01	N/mm
First and second shear fracture energy	3.8	N/mm
Power law coefficient	1	-

The calculations were conducted using a dynamic implicit procedure accounting for geometrical nonlinearity and a time period of 200 s. The boundary conditions and material properties are presented in Section 3.1. In this case, fabric rupture was not observed; hence, the Hashin damage was not defined.

For the carbon fibre textile, the linear quadrilateral membrane elements of type M3D4 was used, and for steel plates, the linear hexahedral elements of type C3D8R was used. The size of the FE mesh set to 0.4 mm consisted of 131,875 elements and 156,744 nodes.

### 3.3. Verification and Validation of Numerical Models

**Figure 15:**
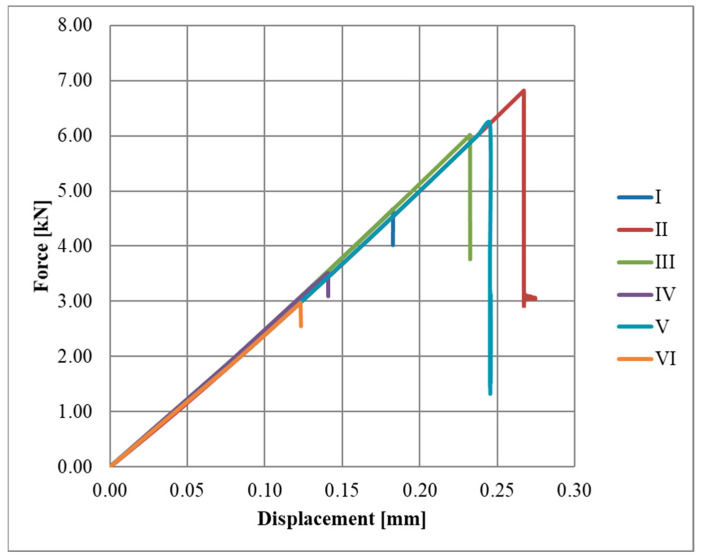
Verification and validation of the fabric rupture model.

In order to verify the effectiveness and quality of the proposed model, validation and verification were carried out (Figure 15). Unfortunately, it was found that the fabric model was very sensitive to the finite element mesh density. With smaller mesh densities, the initiation of destruction occurred faster. It should be emphasised that due to computational limitations, it was not possible to determine a mesh small enough not to affect the calculation result. The smallest tested finite element had a dimension of 0.25 mm.

**Figure 16:**
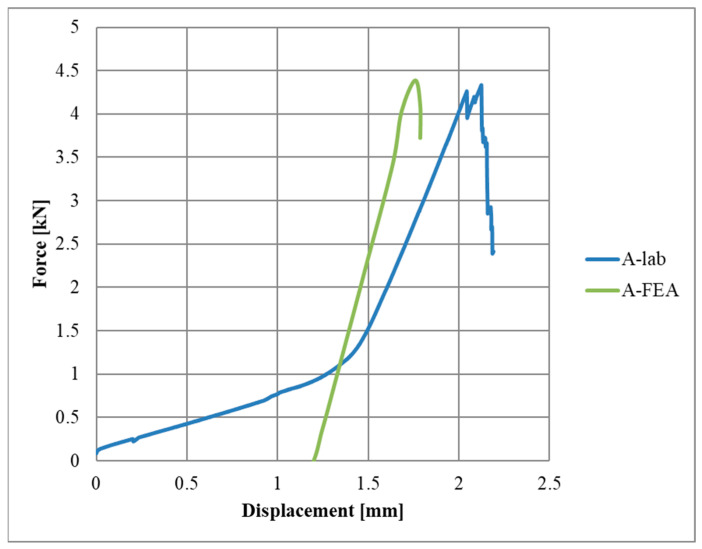
Verification and validation of CZM.

However, in the case of the model for an adhesive connection, the mesh density did not have a significant impact on the obtained results. In common practice, there should be at least five integration points in the thickness of the solid element, and for this reason, the mesh size was set to 0.4 mm. For a smaller mesh value (0.3 mm), the force–displacement graph coincided with the base graph (0.3 mm). Therefore, it can be concluded that the presented model was not sensitive to grid density, which is recommended in engineering practice. The total CPU time for a model with a 0.4 mm mesh size was equal to 4.18 × 10^3^ s. The value of the maximum force was close to that obtained from the laboratory (Figure 16).

## 4. Conclusions

In this paper, the laboratory tests and numerical analysis of a composite bonded joint consisting of galvanised steel elements, an adhesive layer, and CFRP fabric were presented. Different overlap lengths (15 mm, 25 mm, and 35 mm) were taken into account in a shear test conducted on 2 mm thick hot-dip galvanised steel plates of S350 GD overlapped on one side with SikaWrap 230 C carbon fibre textile (CFT) using SikaDur 330 adhesive. Based on the conducted analysis, three forms of joint damage were observed, namely, at the steel–adhesive interface, fibre rupture, and mixed damage behaviour. There was a combination of adhesive failure and fabric rupture, suggesting complex interactions between the materials. It was also noted that most danger adhesive failure occurred at the steel interface at the bond between the steel and adhesive layer. Moreover, it was found that the joint stiffness and strength increased proportionally to the overlap length. A simultaneous increase in adhesive thickness caused a decrease in joint strength. As far the numerical composite model consisting of adhesive and fabric models, unfortunately, the fabric model was very sensitive to the density of the finite element mesh. The smaller the mesh size, the greater the fabric destruction parameters that must be applied. With smaller mesh sizes, the onset of destruction occurred faster. But it should be emphasized that the use of the authors’ own correlation coefficients α and β allowed for the correct mapping of adhesive damage in the numerical model.

## Figures and Tables

**Table 1:** Tested series.

No.	Series Label	Overlap Length (mm)
1	A	15
2	B	25
3	C	35

**Table 2:** Adhesive shear strength test results for the A series.

No.	Sample Label	Maximum Load (kN)	Displacement at Maximum Load (mm)
1	A1	5.60	2.25
2	A2	7.56	2.47
3	A3	4.94	2.04
4	A4	5.88	2.18
5	A5	7.84	2.49
6	A6	6.32	2.56
7	A7	7.74	2.43
8	A8	5.99	2.49
9	A9	8.87	2.75
10	A10	5.17	2.21
11	A11	7.42	2.58
12	A12	5.46	2.32
13	A13	4.91	2.18
14	A14	5.52	2.33
15	A15	5.15	2.26
16	A16	6.03	2.40
17	A17	3.48	2.11
18	A18	4.27	2.12
19	A19	7.91	2.87
20	A20	6.42	2.45
Average		6.12	2.37
Standard deviation		1.386	0.216

**Table 3:** Adhesive shear strength test results for the B series.

No.	Sample Label	Maximum Load (kN)	Displacement at Maximum Load (mm)
1	B1	5.46	2.16
2	B2	5.45	2.12
3	B3	3.84	1.82
4	B4	4.69	2.03
5	B5	6.14	2.30
6	B6	6.97	2.34
7	B7	6.94	2.28
8	B8	5.01	2.03
9	B9	4.75	1.85
10	B10	5.85	2.15
11	B11	4.50	1.98
12	B12	7.45	2.36
13	B13	7.47	2.33
14	B14	2.54	1.63
15	B15	5.70	2.05
16	B16	5.84	2.13
17	B17	4.84	2.04
18	B18	2.40	1.57
19	B19	3.95	1.86
20	B20	4.59	1.97
Average		5.22	2.05
Standard deviation		1.419	0.225

**Table 4:** Adhesive shear strength test results for the C series.

No.	Sample Label	Maximum Load (kN)	Displacement at Maximum Load (mm)
1	C1	8.43	2.27
2	C2	9.98	2.44
3	C3	6.99	2.19
4	C4	7.93	2.29
5	C5	3.07	1.66
6	C6	6.41	2.17
7	C7	7.18	2.25
8	C8	7.10	2.17
9	C9	7.73	2.24
10	C10	5.07	1.90
11	C11	7.62	1.92
12	C12	7.84	2.34
13	C13	8.47	2.16
14	C14	9.55	2.48
15	C15	3.40	1.65
16	C16	9.06	2.59
17	C17	3.04	1.58
18	C18	6.35	2.13
19	C19	6.63	2.07
20	C20	6.30	2.03
Average		6.91	2.13
Standard deviation		1.992	0.273

**Table 5:** Damage mechanisms for each sample.

Sample Label	Damage Mechanism	Sample Label	Damage Mechanism	Sample Label	Damage Mechanism
A1	A, F	B1	A	C1	A, F
A2	F	B2	A	C2	A, F
A3	A	B3	A	C3	F
A4	A, F	B4	A	C4	A, F
A5	A, F	B5	A, F	C5	A
A6	A, F	B6	F	C6	F
A7	A, F	B7	A, F	C7	A, F
A8	A, F	B8	A	C8	A, F
A9	A, F	B9	A	C9	F
A10	A	B10	A, F	C10	F
A11	F	B11	A	C11	F
A12	A, F	B12	F	C12	A, F
A13	A	B13	A, F	C13	A, F
A14	A	B14	A	C14	A, F
A15	F	B15	A	C15	A
A16	A, F	B16	F	C16	A, F
A17	F	B17	A	C17	A
A18	A	B18	A	C18	F
A19	F	B19	A	C19	F
A20	F	B20	A	C20	F

**Table 6:** Average joint strength for each type of failure.

Failure	Series A		Series B		Series C	
	Average Strength [kN]	Standard Deviation [kN]	Average Strength [kN]	Standard Deviation [kN]	Average Strength [kN]	Standard Deviation [kN]
A	4.96	0.41	4.44	0.99	3.17	0.16
F	6.32	1.57	6.75	0.67	6.64	0.79
A, F	6.64	1.13	6.60	0.64	8.39	0.79

**Table 7:** Computer hardware and system type.

Hardware	Type
CPU	12th Gen Intel(R) Core(TM) i5-12500H 2.50 GHz
RAM	64 GB
System	64-bit operating system, x64-based processor

**Table 8:** Carbon fibre textile—elastic material properties.

Parameter	Quantity	Unit
Density	1.83 × 10^−6^	kg/mm^3^
Elastic modulus of fabric E1	220,000	MPa
Elastic modulus of fabric E2	15,750	MPa
Longitudinal and transverse Poisson’s ratio	0.3	[-]
Shear modulus G12	8730	MPa
Shear modulus G13	11,650	MPa
Shear modulus G23	5615	MPa

**Table 9:** Carbon fibre textile—Hashin damage material properties.

Parameter	Quantity	Unit
Longitudinal Tensile Strength	3200	MPa
Longitudinal Compressive Strength	3200	MPa
Transverse Tensile Strength	5	MPa
Transverse Compressive Strength	5	MPa
Longitudinal Shear Strength	5	MPa
Transverse Shear Strength	5	MPa
Fracture Energy	0.01	N/mm

**Table 10:** Mesh sensitivity analysis.

Case	Textile Mesh	Plate Mesh	Number of Elements	Number of Nodes	Hashin Damage Parameters [MPa]	Total CPU Time [s]
I	Linear quadrilateral M3D4	Linear hexahedral C3D8R	70,000	85,951	3	2.92 × 10^5^
II	Linear quadrilateral M3D4	Linear hexahedral C3D8R	10,500	14,637	3	9.49 × 10^3^
III	Linear quadrilateral M3D4	Linear hexahedral C3D8R	70,000	85,951	5	2.75 × 10^5^
IV	Linear quadrilateral M3D4	Linear hexahedral C3D8R	112,000	128,191	5	4.04 × 10^5^
V	Quadratic quadrilateral M3D8R	Linear hexahedral C3D8R	10,500	21,757	5	1.45 × 10^4^
VI	Quadratic quadrilateral M3D8R	Linear hexahedral C3D8R	21,000	53,497	5	4.17 × 10^4^

## Data Availability

The original contributions presented in the study are included in the article, further inquiries can be directed to the corresponding author.
